# Teledentistry—A Futuristic Approach Towards Dental Health Care Delivery: A Cross‐Sectional Study

**DOI:** 10.1155/tswj/5212998

**Published:** 2026-08-07

**Authors:** Shweta Kajjari, Muskaan Adnani, Nithya Annie Thomas, Vanishree B. K., Hema Kanathila, Vidyavathi H. Patil

**Affiliations:** ^1^ Department of Pediatric and Preventive Dentistry, KLE V.K. Institute of Dental Sciences, KLE Academy of Higher Education and Research, Belagavi, Karnataka, India; ^2^ Department of Pediatric and Preventive Dentistry, Manipal College of Dental Sciences, Manipal Academy of Higher Education, Manipal, Karnataka, India, manipal.edu; ^3^ Department of Pediatric and Preventive Dentistry, M.A.Rangoonawala College of Dental Science and Research Centre, Pune, Maharashtra, India; ^4^ Department of Prosthodontics and Crown and Bridge, KLE V.K. Institute of Dental Sciences, KLE Academy of Higher Education and Research, Belagavi, Karnataka, India

**Keywords:** attitude, dental professionals, perception, practice, teledentistry

## Abstract

**Background:**

With the advent of telecommunication services, dental consultation has advanced from the mere use of telephones to the use of digital imaging and the internet to provide consultation, exchange health information, and maintain electronic health records beyond borders for increased efficiency of dental healthcare delivery to patients.

**Aim:**

To assess dental professional′s perception, attitude, and practice of teledentistry in rural areas of Belagavi district, Karnataka, India.

**Methodology:**

A cross‐sectional survey comprising of 450 participants was conducted among dental professionals, which included dental interns, postgraduate students, faculty members of dental colleges, consultants, and private practitioner who works at rural areas of Belagavi district. A 16‐item validated questionnaire containing four domains of professional demographic data, perception, attitude, and practices was distributed among the participants. Descriptive analysis was done using IBM SPSS software (Version 20.0, Chicago, Illinois, United States). The level of significance was set at *p* < 0.05.

**Results:**

It was found that 52% of dental professionals had low levels of perception. However, levels of attitude and practice were found to be high. When correlation between perception, attitude, and practice was carried out using Pearson′s correlation coefficient, a positive correlation was seen between attitude and practice (*p* = 0.0001^∗^).

**Conclusion:**

The majority of dental professionals exhibited low perception levels but demonstrated high levels of attitude and practice. The effective implementation of teledentistry could usher in a new era of dental healthcare delivery, improving oral health among rural populations.

## 1. Introduction

The prevalence of poor oral hygiene is widespread in India. It is well recognized that inadequate oral hygiene is more common in rural areas than in urban areas due to factors such as limited awareness, financial constraints, restricted access to dental facilities, excessive consumption of refined sugars, neglect of oral hygiene practices, and habits such as smoking and smokeless tobacco use. In addition, the healthcare sector faces significant public health challenges driven by population growth and aging, including increased demand for healthcare services, workforce shortages, limited access to care, and prolonged waiting times [1, 2]. These challenges can further limit access to oral healthcare services, particularly in underserved rural communities. Addressing such issues requires substantial transformation of healthcare systems, as rising healthcare expenditures and existing service models are becoming increasingly unsustainable over the long term [2, 3]. Consequently, there is a growing need for cost‐effective innovations that enhance efficiency, reduce pressure on healthcare systems, and maintain the quality of care. Digital technologies offer valuable opportunities to improve healthcare delivery, increase operational efficiency, and support the provision of high‐quality, cost‐effective healthcare, including oral healthcare services.

In the year 2020, the COVID‐19 pandemic spread worldwide, significantly impacted the healthcare system and created a global healthcare concern that adversely affected the lifestyles of both patients and dentists. In addition, mandatory social distancing measures were introduced due to the high risk of infection for both patients and health care professionals. The COVID‐19 pandemic led to a paradigm shift towards the development of virtual e‐dentistry programs through the introduction of user‐friendly technologies, thereby providing an effective foundation for sustainable dental care while prioritizing risk reduction for both patients and dentists [4, 5].

This situation familiarized the concept of using the internet for dental consultations, known as teledentistry. Teledentistry is “the practice of using video‐conferencing technologies to diagnose and provide advice about treatment over a distance [6].” Furthermore, the International Association of Dental Research (IADR) and the terminology consensus report defines teledentistry as follows: “Teledentistry represents the uses of information and telecommunication technology to provide oral healthcare services between an oral healthcare provider and a patient/recipient or other health care providers who are separated by distance [7].”

The pandemic made clear the importance of teledentistry—previously a largely overlooked concept—as healthcare providers increasingly applied telecommunications for patient care [8]. Teledentistry is an emerging trend with the combination of dentistry and telecommunication [9]. Teledentistry centers on “dental triage,” encompassing the prompt alleviation of pain or infection, remote delivery of dental care through consultations, and the strategic planning and scheduling of comprehensive dental treatments. According to a systematic review, teledentistry is a valid tool comparable to face‐to‐face consultations for oral disease identification, accurate diagnosis, and teleconsultation services. Furthermore, electronic specialist referrals can be facilitated without placing an undue burden on frontline healthcare professionals [10].

“Additional applications of teledentistry encompass its role in dental education. This encompasses two primary modalities: web‐based self‐instruction and interactive video conferencing, which are used to educate dental students. Furthermore, teledentistry serves as a valuable tool for continual dental education among professionals, allowing them to stay abreast of advancements in the field of practice.” In the field of Pediatric and Preventive Dentistry, there is an urge for schools and childcare centers to adopt teledentistry for early detection of oral diseases before they progress into emergencies. This approach promotes safe oral hygiene among children, enables timely management of emerging oral problems, and connects children and their parents with appropriate health and social services [11]. Teledentistry is a valuable assessment tool for early childhood caries and identifying patients based on their risk levels, which are high or low [12].

The findings of systematic review and meta‐analysis suggest that teledentistry offers significant benefits for patients, oral health care professionals, other health care providers, and for the public domain. However, several studies have also highlighted implementation challenges such as the absence of clear policies and guidelines, inadequate training, and limited knowledge among health care professionals and digital literacy [13]. For patients residing in rural or remote areas, where traveling to and from a dental clinic poses a major challenge, teledentistry utilization offers a promising and practical solution [14, 15]. Considering the increasing utilization of teledentistry, the challenges faced by healthcare professionals, and the existing gaps in the evolving knowledge regarding teledentistry, the present study was conducted to assess the perception, attitude, and practice about teledentistry among dental professionals in the rural areas of Belagavi—a district in Karnataka, India—where the population stands at approximately 5,305,424 out of which the rural population is approximately 67.26% (3,568,466) [16].

## 2. Materials and Methods

This study was a cross‐sectional questionnaire study conducted among dental interns, postgraduate students, and faculty members of dental colleges, consultants, and private practitioners who work in the rural areas of Belagavi, Karnataka, India. Ethical approval was obtained from the Institutional Research and Ethics Committee, KLE VKIDS, Belagavi. Sample size of 458 was calculated using the standard sample size formula,

n=Z1−a/2+Z1−β2 p1q1+p2q2p1−p22,

(where *p*_1_ = 0.57, *p*_2_ = 0.67, q1 = 0.43, q2 = 0.33, *Z*_1−(*a*/2)_ = Alpha error at 95*%* = 1.96, *Z*_1−*β*_ = Beta error at 85% power = 1.03) and was rounded to 450 [17].

The questionnaire used in the present study was adapted from a teledentistry cross‐sectional survey originally developed by Mandall et al. [18] and later modified by Estai et al. [19]. The revised version was considered more appropriate for assessing dentists′ overall perspectives on teledentistry, rather than confining the evaluation to specific specializations alone. Accordingly, the present study adopted the modified questionnaire developed by Estai et al., which consists of four domains encompassing a total of 16 questions. “The first domain asked the participants to provide their demographic data, the second dealt with the perception of dental professionals regarding teledentistry, the third dealt with the attitude, and the last dealt with the practices.” The response to the questions was measured on a four‐point Likert scale: “Strongly disagree,” “Disagree,” “Strongly agree,” “Agree”. Likert scale is a psychometric scale commonly used in research contexts to measure attitudes or opinions or perceptions [20]. A four‐point Likert scale was used in the questionnaire, with no neutral option, so respondents must choose a specific response [21]. The content validation of the questionnaire was done by a peer review team. A preliminary study was evaluated among 15 health care professionals for clarity, transparency, and ease of understanding of the questionnaire. Reliability of those questionnaires was assessed and a “Cronbach alpha coefficient” value of 0.82 was obtained. These study samples were then excluded in the present final study; the questionnaires neither required any corrections nor modifications, and it was well accepted. The inclusion criteria for the study were interns, postgraduate students, faculty members of dental colleges, consultants, and private practitioners who were working in the rural areas of Belagavi district. The exclusion criteria for our study were the undergraduate students and those who did not give their consent to participate.

The link of the Google form questionnaire was sent via WhatsApp/Emails among 450 participants. Upon the completion of the survey, the respondents were instructed to submit the Google form. Reminder messages were sent to all the participants every 3 days until the submission of the filled questionnaire form. The identity of the participants was kept confidential. The data were then entered in MS excel sheet (Microsoft Corp.) and were analyzed using IBM SPSS software (Version 20.0 Chicago Illinois, United States). Chi‐square, one way ANOVA, and Karl Pearson′s correlation coefficient methods were used for statistical analysis. “*p* < 0.05” was considered statistically significant.

## 3. Results

A total of 450 participants were included in the study, comprising varied age groups, years of experience, gender, and designations. Of these, 26.22% were males and 73.78% were females. The mean age of the participants was 26.59 ± 9.02 years. Fifteen questions with a wide assortment assessed their perception, attitude, and practice level (Table 1 and Table 2).

**Table 1:** Mean percentage of responses of respondents on questions related to perception among dental professionals.

Questions on perception	No. of dental professionals	% of dental professionals
Q1—The applicability of teledentistry is more about:		
Time savings	238	52.89
Cost effectiveness	165	36.67
Access to underserved	222	49.33
Enhanced communication of oral healthcare needs	275	61.11
Q2—Do you think teledentistry is		
i. A valuable tool in dentistry		
ii. About providing higher comfort level for dentists and patients		
iii. Learning new methods of care delivery		
iv. Limited value in emergency		
i, ii, iii	165	36.67
ii, iii, iv	132	29.33
i, iii, iv	170	38.00
i, ii, iii, iv	333	74
Q3—Which one of the following do you think is the best means of communication for teledentistry?		
Plain old telephone system	54	12.00
Integrated services digital network	224	49.78
World wide web based teledentistry	166	36.89
Emails	0	0.00
Q4—Which of these do you think is a major challenge for teledentistry in rural parts of India?		
Illiteracy	109	24.22
Lack of awareness	203	45.11
Poverty	36	8.00
Lack of infrastructure	102	22.67
Q5—Which of these methods will be helpful to promote teledentistry in rural areas?		
Community education	333	74.00
Posters	171	38.00
Pamphlets	132	29.33
Radio	165	36.67

**Table 2:** Mean percentage of responses of respondents on questions related to attitude and practice among dental professionals.

Attitude and practice questions	Strongly disagree	%	Disagree	%	Agree	%	Strongly agree	%
Q6—Do you think information received over teledentistry is sufficient to formulate a final diagnosis and treatment plan?	28	6.22	200	44.44	156	34.67	66	14.67
Q7—Do you think teledentistry can be used to bring awareness about oral health care to the rural population?	0	0.00	19	4.22	299	66.44	132	29.33
Q8—Do you think teledentistry can increase patient inflow in rural areas?	0	0.00	34	7.56	317	70.44	99	22.00
Q9—Do you agree that the dentist will confer with a specialist about the treatment through teledentistry?	0	0.00	39	8.67	339	75.33	72	16.00
Q10—Do you think the government should take an initiative to promote teledentistry in rural areas?	0	0.00	12	2.67	293	65.11	145	32.22
Q11—Do you prefer to use teledentistry for routine check‐ups and follow‐ups?	10	2.22	84	18.67	272	60.44	84	18.67
Q12—Do you think additional skills are required by a practitioner to include teledentistry in day‐to‐day practice?	0	0.00	90	20.00	297	66.00	63	14.00
Q13—Do you think teledentistry can be helpful to patient in receiving interdisciplinary medical care?	3	0.67	39	8.67	330	73.33	78	17.33
Q14—Do you believe teledentistry will be helpful in resolving clinical emergencies by an amateur dentist?	48	10.67	102	22.67	252	56.00	48	10.67
Q15—Do you prefer sending e‐prescriptions to the patient?	16	3.56	96	21.33	288	64.00	50	11.11
Q16—Will you encourage your colleagues to practice teledentistry?	0	0.00	30	6.67	333	74.00	87	19.33

**Figure 1:**
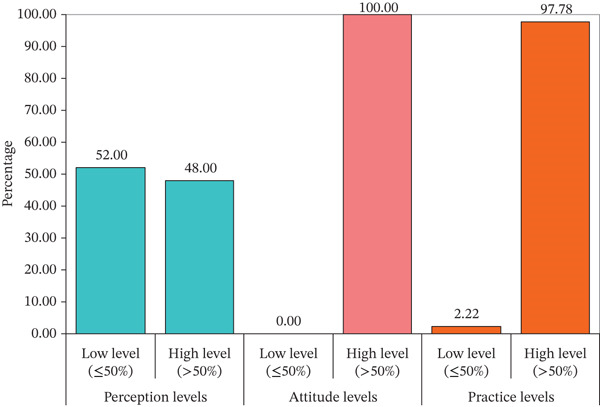
Distribution of dental professionals with levels of perception, attitude, and practice.

In the results, for the clear illumination “agree + definitely agree” and “disagree + definitely disagree” were combined. 52% of dental professionals had low levels of perception; on the contrary, levels of attitude and practice were found to be high (Figure 1).

When the levels of perception were compared across age groups, participants aged 29 years or less demonstrated a higher level of perception than those in older age groups, and this difference was statistically significant (*p* = 0.0001^∗^). With respect to gender, although females showed better perception than males, the difference was not statistically significant. When perception was assessed based on other demographic variables such as designation and years of experience, interns, postgraduates, and those with less than 1 year of experience showed higher levels of perception. Statistically significant differences were observed for age group (*p* = 0.0001^∗^), designation (*p* = 0.003^∗^), and years of experience (*p* = 0.0001^∗^), respectively (Table 3).

**Table 3:** Association between levels of perception with demographic profile.

Profile	Low level	%	High level	%	Total	Chi‐square	*p*‐value
Age groups							
20‐29 years	188	48.33	201	51.67	389	15.4990	0.0001 ^∗^
30‐39 years	19	76.00	6	24.00	25		
≥ 40 years	27	75.00	9	25.00	36		
Gender							
Male	67	56.78	51	43.22	118	1.4640	0.2260
Female	167	50.30	165	49.70	332		
Designation							
Interns	136	49.10	141	50.90	277	15.9930	0.0030 ^∗^
Postgraduate	46	46.00	54	54.00	100		
Private practitioner	15	71.43	6	28.57	21		
Consultant	6	50.00	6	50.00	12		
Faculty	31	77.50	9	22.50	40		
Experience							
0–1 year	155	48.44	165	51.56	320	15.8270	0. 0001 ^∗^
2–5 years	35	49.30	36	50.70	71		
6–10 years	16	64.00	9	36.00	25		
> 10 years	28	82.35	6	17.65	34		
Total	234	52.00	216	48.00	450		

*Note:* % represents percentage.

^∗^ *p* < 0.05.

When the levels of attitude with respect to varied age group, gender, designation and years of experience were compared, it showed better attitudes among all the dental professionals.

The levels of practice across age groups showed that participants in the younger age group (20–29 years) demonstrated significantly better levels of practice compared to the older age groups, with a statistically significant *p*‐value (0.0001 ^∗^). With respect to gender, females exhibited higher level of practice than males, and this difference was also statistically significant (*p* = 0.0010^∗^).When the association between levels of practice and designation was examined, the results indicated that interns and consultants demonstrated the highest level of practice, whereas postgraduates, faculty members, and private practitioners exhibited comparatively lower practice level. This association was also found to be statistically significant (*p* = 0.0001^∗^). Regarding years of experience, dental professionals with less than 10 years of experience showed markedly higher levels of practice, whereas those with more than 10 years of experience had lower practice levels. This exhibited a statistically significant difference of *p* = 0.0001^∗^ (Table 4). When the comparison of designations with mean perception, attitude, and practice scores was analyzed, it revealed statistical significant results with the *p*‐value of 0.0001 ^∗^, respectively (Table 5). When Pearson′s correlation coefficient was assessed between perception, attitude, and practice, positive correlation was seen between attitude and practice, which was statistically significant (*p* = 0.0001^∗^) [Table 6].

**Table 4:** Association between levels of practice with demographic profile.

Profile	Low level	%	High level	%	Total	Chi‐square	*p*‐value
Age groups							
20–29 years	3	0.77	386	99.23	389	31.7980	0.0001 ^∗^
30–39 years	4	16.00	21	84.00	25		
≥ 40 years	3	8.33	33	91.67	36		
Gender							
Male	7	5.93	111	94.07	118	10.1310	0.0010 ^∗^
Female	3	0.90	329	99.10	332		
Designation							
Interns	0	0.00	277	100.00	277	32.0480	0.0001 ^∗^
Postgraduate	3	3.00	97	97.00	100		
Private practitioner	3	14.29	18	85.71	21		
Consultant	0	0.00	12	100.00	12		
Faculty	4	10.00	36	90.00	40		
Experience							
0–1 year	3	0.94	317	99.06	320	57.3940	0.0001 ^∗^
2–5 years	0	0.00	71	100.00	71		
6–10 years	0	0.00	25	100.00	25		
> 10 years	7	20.59	27	79.41	34		
Total	10	2.22	440	97.78	450		

*Note:* % represents percentage.

^∗^ *p* < 0.05.

**Table 5:** Comparison of designations with mean perception, attitude, and practice scores by one way ANOVA.

Designations	Perception			Attitude			Practice		
	Mean	SD	SE	Mean	SD	SE	Mean	SD	SE
Interns	6.30	2.22	0.13	15.52	1.83	0.11	17.95	2.25	0.14
Postgraduate	6.32	1.70	0.17	14.86	1.66	0.17	16.90	2.46	0.25
Private practitioner	5.14	1.01	0.22	13.71	0.90	0.20	14.86	2.29	0.50
Consultant	6.75	2.60	0.75	16.50	2.15	0.62	19.00	4.49	1.30
Faculty	5.08	1.49	0.24	15.50	2.57	0.41	17.83	3.59	0.57
Total	6.15	2.06	0.10	15.32	1.90	0.09	17.59	2.62	0.12
*F*‐value	4.9162			7.6760			10.4960		
*p* value	0.0001 ^∗^			0.0001 ^∗^			0.0001 ^∗^		

Abbreviations: SD, standard deviation; SE, standard error.

^∗^ *p* < 0.05.

**Table 6:** Correlations among perception, attitude, and practice scores by Karl Pearson′s correlation coefficient.

Variables	Correlation coefficient	*t*‐value	*p*‐value
Perception and attitude scores	−0.0589	−1.2488	0.2124
Perception and practice scores	−0.0653	−1.3844	0.1669
Attitude and practice scores	0.7562	24.4645	0.0001 ^∗^

^∗^ *p* < 0.05 indicates significant correlation.

The findings of the present study indicate that younger dental professionals and those with lesser years of experience demonstrated greater acceptance and utilization of teledentistry. These results highlight the growing importance of digital literacy and technology‐based healthcare delivery in dental practice. Furthermore, the positive correlation between attitude and practice suggests that favorable attitudes towards teledentistry may enhance its practical implementation in clinical settings. The study findings may have important implications for clinical practice, dental education, and healthcare policy, particularly in improving accessibility and promoting the integration of teledentistry services in rural areas.

## 4. Discussion

Since the COVID‐19 pandemic, the use of telehealth has significantly expanded including various medical services delivered through electronic communication technologies to improve disease management and healthcare accessibility. Telehealth is cost‐effective and benefits underserved communities. Teledentistry, a subset of telehealth, similarly enhances dental care access. Its importance has been highlighted during the pandemic for providing remote dental care services without disease transmission risks. Though not new, COVID‐19 has underscored teledentistry′s vital role in delivering a range of dental services remotely, benefiting overall health.

The American Dental Association outlines four teledentistry modalities: live‐video monitoring (synchronous), store‐and‐forward (asynchronous), remote patient monitoring (RPM), and mobile health (m‐Health) [22]. Synchronous involves real‐time, two‐way interactions, often via video. Asynchronous entails sending data (e.g., radiographs, images) for remote evaluation. Messenger apps like WhatsApp are commonly used for synchronous interactions due to their user‐friendly interface, along with video conferencing apps and smart phone tools for data transmission [23].

Current study collected data from dental professionals to assess their perception, attitude, and practice of teledentistry across rural areas of Belagavi district, Karnataka, India. As the literature concerns, this was the largest survey in Belagavi district, Karnataka, to explore dental professionals′ perceptions of the application of teledentistry in routine dental practice.

The previous literature when compared with the present results indicated that perception about teledentistry differs by country, designation, and years of experience [24–27]. Current study revealed that the majority of dental professionals generally lack perception about integrating teledentistry into daily practice. Interns and postgraduates exhibited a higher level of perception and were more enthusiastic about teledentistry compared to consultants, faculty members, and private practitioners, who showed relatively lower levels of perception. Possible reasons may include lack of education, rapid technological changes, limited awareness, resistance to change, complex regulations, and resource constraints. Notably, dental professionals aged 29 years or younger had better knowledge compared to older ones, possibly due to more recent education or the digital era, which aligns with previous research done by Save et al. [28], Subhan et al. [29], and Lin et al. [30]. The study also revealed that, despite experience, the majority of the dental professionals had low perception levels regarding teledentistry′s application in daily practice. Years of experience and age exhibited an inverse relationship with the perception level about teledentistry, which is consistent with the findings of the World Health Organization [2]. These challenges act as barriers to the effective integration of teledentistry.

Present study evaluated the practice and attitude levels of dental professionals regarding the teledentistry; it revealed that in spite of lack of perception, majority of the dental professionals had good attitude and practice towards teledentistry usage and applications. The current study found that majority of dental professionals urge an intention to use teledentistry as a part of the practice in the future, this might be due to aiming to enhance efficiency, improve access for underserved populations, elevate the quality of care, and reduce the burden of oral diseases. Similar findings were reported with the study done by Alshammari et al. [31].

Most of the dental practitioners at rural areas accepted the fact that teledentistry can enhance communication of oral healthcare needs, is cost‐effective, and saves time for the patient and the dentist. This is in accordance with the studies done by Eraso et al. [32], Yoshinaga et al. [33], and Estai et al. [34].

In the present study, most participants preferred Integrated Services Digital Network (ISDN) for teledentistry communication in favour of email and phone. Dental professionals in the present survey preferred social media, notably WhatsApp, similar to findings in Saudi Arabia [35]. Three‐fourths believed teledentistry could provide accurate information and specialist care to rural areas and offered a convenient examination method [9]. Proficient teledentistry typically requires a smart phone and internet access, which are commonly available in dental clinics, making its integration feasible.

Our results indicated that 45% of respondents identified a lack of awareness as the primary challenge for teledentistry in rural India. Additionally, the perception that teledentistry requires dental professionals to acquire technical skills may contribute to hesitation in this field. A study by Save et al. [28] indicated that teledentistry demands moderately complex technical skills, but 41.1% believed these skills would be relatively simple.

When participants were surveyed to ascertain their perspectives regarding the efficacy of various strategies for advancing the adoption of teledentistry in rural regions, 74% of participants mentioned that community education plays a pivotal role in achieving this goal.

When asked about teledentistry′s potential to raise oral healthcare awareness among rural populations, 97% of respondents recognized its value. In India, where a majority (68.84%) lives in rural areas [36] and only 10% of dental professionals serve these underserved regions, swift diagnosis and treatment are essential [37]. Limited awareness and education contribute to rural neglect of oral health [38]. Lin et al.′s meta‐analysis during the COVID‐19 pandemic found that dental professionals had a higher awareness and positive attitude towards teledentistry. Additionally, 92% believed teledentistry could increase patient flow in rural areas [30].

About 91.33% of respondents agreed that teledentistry could facilitate consultations between dentists and specialists on behalf of patients. Dentistry encompasses various specialties, and sometimes, general dentists may lack the expertise to diagnose and plan treatment accurately. Teledentistry can bridge this experiential gap, enabling less‐experienced dentists to consult experts, thus improving oral healthcare quality. This aligns with Save et al.′s study, where 86.8% believed teledentistry could enhance diagnosis [19]. In emergencies or complex cases, teledentistry offers valuable guidance from specialists.

Approximately, 97.33% believed that the government should promote teledentistry in rural areas. Government support can optimize resource allocation, inform policies, enhance health education, build telehealth infrastructure, and recognize oral health′s importance in overall well‐being. About 79.11% of participants expressed a preference for using teledentistry for routine checkups and follow‐ups, indicating openness to this approach over traditional in‐person visits.

Approximately 80% of respondents acknowledged the need for additional skills to integrate teledentistry into daily practice. This involves mastering telecommunication tools, video conferencing, digital platforms, technical troubleshooting, maintaining secure digital records, and effective virtual communication. Integrating teledentistry can enhance access to care, patient engagement, and convenience, but dentists must acquire the necessary skills to navigate the unique challenges and opportunities of remote dental care effectively.

About 90.66% of respondents agreed that teledentistry can facilitate interdisciplinary medical care, allowing dental healthcare providers to collaborate with other medical specialists. This collaboration streamlines patient care coordination, enhances diagnostic accuracy, identifies underlying conditions for timely intervention, and improves patient education. The holistic approach benefits those with complex health needs.

75.11% preferred e‐prescriptions, aligning with Zimamu et al.′s [39] and Jariwala et al.’s [40]studies, where physicians also recognized its benefits, including time‐saving, safety, and improved patient services. 93.33% of respondents would support their colleagues in adopting teledentistry, signaling strong enthusiasm within the dental community. This positive attitude may boost the broader acceptance and integration of teledentistry in dental practices.

One of the most noteworthy findings of the present study is the lack of perception due to formal training concepts about teledentistry among dental professionals. This absence of formal education in teledentistry highlights a significant gap in dental curricula and emphasizes the necessity for targeted training programs to equip dental professionals with the skills needed to effectively incorporate digital tools into their practice [41]. Further emphasizing the willingness of dental professionals to adopt new technologies, provided they receive adequate support and training. As the acceptance of teledentistry in the present study was largely influenced by knowledge and familiarity, the incorporation of structured training modules into undergraduate curricula and continuing dental education programs is essential. Previous evidence has demonstrated that targeted educational interventions can positively influence attitudes towards teledentistry and enhance confidence in its clinical application [42]. Such training programs may include case‐based learning, practical exposure to store‐and‐forward systems, and interdisciplinary consultation exercises addressing data protection requirements and diagnostic limitations. Furthermore, the integration of digital and tele‐education approaches into dental curricula has been shown to improve learning outcomes, clinical skill development, and professional confidence in the use of information and communication technologies, thereby supporting the effective adoption of teledentistry in routine dental practice [43, 44]. Recent implementation studies have further emphasized the need for clearly defined learning objectives, appropriate delivery methods, and robust assessment strategies to ensure sustainable and effective teledentistry training [45].

Dental treatment requires highly specialized equipment and technique‐sensitive procedures. Incorporating teledentistry to National Health Care System enables the underprivileged communities to avail oral health care especially at the Primary Health Care centers, which might have a significant preventive impact by spreading awareness about oral diseases and importance of proper oral hygiene (including the using of web based self‐instruction and/or video conferencing tools). Additionally, teledentistry can be used to remotely deliver specialized clinical training to dental health care professionals working in beneficiary areas [46].

In developing countries like India, each district hospital should also establish telecommunication links with a remote panel of super specialists who can be consulted for additional treatment plans or guidance as needed. For the successful implementation of the teledentistry initiative, it is essential to collaborate with an internet service provider (ISP) that can support the program by offering free or subsidized bandwidth as part of its corporate social responsibility (CSR) efforts [47].

Our study findings indicate that health policies should prioritize comprehensive training for dental professionals to strengthen their expertise and practical application of teledentistry. By focusing on advanced education, clinicians can make more informed decisions in patient management and effectively implement teledental practices, ultimately resulting in greater patient satisfaction. Additionally, it is essential to establish an institutional framework and a clear policy agenda that addresses key concerns such as practitioner liability, patient protection against malpractice, safeguarding patient data privacy, and obtaining informed consent in advance.

## 5. Limitations

In our investigation, certain limitations should be considered. First, our study focused primarily on dental professionals′ perspectives regarding the adoption of teledentistry, and it did not include in‐depth assessments of the infrastructure readiness of Indian dental hospitals or clinics for teledentistry practice for its practical implementation. Second, although we identified concerns related to the ability to make accurate diagnoses, our study did not delve deeply into the specific barriers or mechanisms hindering the broader integration of teledentistry into oral health services nationwide. Furthermore, the possibility of self‐selection bias cannot be excluded, as dental professionals with a greater interest in digital technologies may have been more likely to participate in the study. This may have influenced the representativeness of the findings, particularly regarding the perceived barriers and concerns associated with teledentistry. In addition, owing to the cross‐sectional nature of the study, the findings reflect only the current perceptions and practices of the participants at a single point in time. Therefore, longitudinal studies are warranted to evaluate changes in perceptions and utilization of teledentistry over time with advancements in experience, infrastructure, and healthcare policies. Finally, to acknowledge that patients′ perspectives on teledentistry remain an essential area for further research.

## 6. Clinical Significance

Present study has contributed to the understanding of teledentistry from the perspective of dental professionals. Yet, there is a need for further research, including studies examining teledentistry usage from both patients′ viewpoints and dentists′ knowledge and practices in India. Teledentistry holds significant promise for implementation across various dental areas, depending on patients′ treatment needs. It offers an effective approach to enhance patient dental care by reducing waiting times and treatment delays.

## 7. Futuristic Implications

A pivotal consideration involves pinpointing the most suitable dental specialties for integrating teledentistry, supported by the creation of guidelines and a legal/ethical framework that guarantees standardized dental care while preserving patient privacy. Evolving dental curricula must align with contemporary practice needs, necessitating dental professionals to stay updated and equipped for evolving oral care demands. To address these challenges and promote teledentistry, a focus on education, regulation, and organizational integration is key. This involves integrating teledentistry into dental programs, providing resources for professionals, clarifying regulations, and incentivizing teledentistry through reimbursement policies and professional associations′ support.

Artificial intelligence (AI) has evolved tremendously in recent years, and the application of AI in teledentistry has the potential to revolutionize remote dental care. Machine learning, including deep learning‐based algorithms, has been developed to create predictive models of risk assessment and diagnostic services for oral health, which can enable teledentistry to better its remote screening, diagnoses, record keeping, triaging, and monitoring of dental diseases [48]. Therefore, in the future, teledentistry with the integration of AI can play a bigger role in improving efficiency and quality of care, and support healthcare systems providing cost‐effective care. To reach this objective, research will also need to be stepped up in this AI area so that cost‐effective interventions can be implemented.

## 8. Conclusion

The majority of dental professionals exhibited low perception levels but demonstrated high levels of attitude and practice. Hence, there is a need to implement the knowledge and promotions of teledentistry among dental professionals. Integrating teledentistry into education and training programs could enhance overall knowledge levels. Further, multicentric studies with larger sample sizes and wider geographic representation are recommended to better evaluate the perception, attitude, and practice of teledentistry among dental professionals. Effective implementation of teledentistry could usher in a new era of accessible, efficient, and patient‐centered dental health‐care delivery, particularly for rural communities.

## Author Contributions

Dr Shweta Kajjari contributed to conceptualization including the design of the protocol. Dr Muskaan Adnani contributed the database and carried out the study. Dr Shweta Kajjari had full access to all of the data in the study and takes complete responsibility for the integrity of the data and the accuracy of the data analysis.

## Funding

No funding was received for this manuscript.

## Disclosure

All authors have read and approved the final version of the manuscript.

## Conflicts of Interest

The authors declare no conflicts of interest.

## Data Availability

The data that support the findings of this study are available on request from the corresponding author. The data are not publicly available due to privacy or ethical restrictions.
